# Improved Preventive Effects of Combined Bioactive Compounds Present in Different Blueberry Varieties as Compared to Single Phytochemicals

**DOI:** 10.3390/nu11010061

**Published:** 2018-12-29

**Authors:** Simone G. J. van Breda, Jacob J. Briedé, Theo M. C. M. de Kok

**Affiliations:** Department of Toxicogenomics, GROW School for Oncology and Developmental Biology, Maastricht University, P.O. Box 616, 6200 MD Maastricht, The Netherlands; j.briede@maastrichtuniversity.nl (J.J.B.); t.dekok@maastrichtuniversity.nl (T.M.C.M.d.K.)

**Keywords:** disease risk reduction, blueberry extracts, (combinations of) phytochemicals, antioxidant capacity, gene expression profiles

## Abstract

Blueberries contain many different phytochemicals which might be responsible for their disease preventive properties. In a previously conducted human dietary intervention study, we showed that a 4-week intervention with blueberry–apple juice protected the participants against oxidative stress and modulated expression of genes involved in different genetic pathways contributing to the antioxidant response. The present study investigates the effect of different blueberry varieties (Elliot, Draper, Bluecrop, and Aurora, and the blueberry–apple juice from our previous human dietary intervention study), and four different single compounds (vitamin C, peonidin, cyanidin, and quercetin) on antioxidant capacity and gene expression changes in colonic cells in vitro, and compares the outcome with the earlier in vivo findings. The results demonstrate that all blueberry varieties as well as the blueberry–apple juice were more effective in reducing oxidative stress as compared to the single compounds (e.g., DNA strand break reduction: EC_50_: Elliot 8.3 mg/mL, Aurora and Draper 11.9 mg/mL, blueberry–apple juice 12.3 mg/mL, and Bluecrop 12.7 mg/mL; single compounds). In addition, the gene expression profiles (consisting of 18 selected genes from the in vivo study) induced by the blueberry varieties were more similar to the profile of the human intervention study (range 44–78%). The blueberry variety Elliot showed the strongest and most similar effects, almost 80% of gene expression modulations were similar compared to the in vivo results. From the single compounds (range 17–44%), quercetin induced the most comparable gene expression changes, i.e., 44%. This approach could be useful in agriculture for identifying crop varieties containing combinations of phytochemicals which show optimal preventive capacities.

## 1. Introduction

Consumption of fruits and vegetables has generally been associated with a decreased risk of various chronic diseases including different types of cancer, diabetes, and cardiovascular illnesses. Over the years, numerous bioactive compounds have been identified that may contribute to these beneficial health effects [1]. More recently, evidence is emerging that specific combinations of phytochemicals may be far more effective in protecting against cancer as compared to the effects of isolated compounds due to synergistic interactions [2,3,4]. Although our understanding of the molecular mechanisms underlying potential synergistic effects is still limited, it appears that different combinations of complementary modes of actions are involved, each induced by separate compounds in the mixture. The complexity of such mechanisms is demonstrated by a number of proof-of-principle studies with combinations of vegetables [5,6,7] and fruits [8]. Furthermore, in a study testing a blueberry–apple juice combination it was shown that complex whole genome gene expression responses that are associated with decreased induction of oxidative DNA damage, can be linked to crucial processes in cancer development as well as other chronic diseases [9,10,11].

These findings imply that disease prevention strategies based on whole diet interventions are likely to be more effective as compared to the use of dietary supplementation with isolated single compounds. Furthermore, understanding the relevance of multiple molecular mechanisms induced by combinations of bioactive compounds may allow for the improvement of such strategies beyond the level of just maximizing only one specific characteristic, e.g., total antioxidant potential.

In this study, we therefore focus on the biological effects induced by different blueberry varieties, known to have different antioxidant capacities. As a follow-up to our previous human dietary intervention study with a blueberry–apple juice, we now evaluate the capacity of this blueberry–apple juice as well as different blueberry extracts to induce gene expression changes in cultured human colonocytes (Caco-2 cells) focusing on a selected subset of 18 relevant genes from the in vivo study. The selected genes play a key role in identified biological processes, such as immune response, lipid metabolism, cell adhesion, and apoptosis, which contribute to the antioxidant response of blueberry–apple juice [9,10]. Furthermore, specific endpoints for (anti)oxidant capacity such as the quantification of the induction of DNA strand breaks after a challenge with tert-butylhydroperoxide and the formation of reactive oxygen species are established. Additionally, we compare the effects with those induced by four single compounds, vitamin C, peonidin, cyaniding, and quercetin, all known contributors to the antioxidant value of blueberries and present in different concentrations in the different varieties. We hypothesize that complex mixtures of phytochemicals as present in the blueberry extracts, show a higher capacity to reduce reactive oxygen species ROS generation and DNA damage and that the induced gene expression changes resemble the in vivo induced profile more similarly than the single compounds. It is also expected that the blueberry extract that is relatively high in antioxidants will show a more pronounced effect on ROS formation, DNA damage, and gene expression changes. To the best of our knowledge, this is the first study using gene expression profiles established in humans to characterize biological effects induced by different food products, in vitro. 

## 2. Material and Methods

### 2.1. Cell Culture

The human colonic adenocarcinoma cell line Caco-2 was cultured in Dulbecco’s Modified Eagle’s Medium (Sigma-Aldrich, Zwijndrecht, The Netherlands) with 4.5 g/L glucose, L-glutamine, NaHCO_3_, and pyridoxine HCl supplemented with 10% (*v*/*v*) heat-inactivated fetal bovine serum, 1% (*v*/*v*) non-essential amino-acids, 1% (*v*/*v*) sodium pyruvate, and 1% (*v*/*v*) penicillin/streptomycin (Gibco, Breda, The Netherlands). Cell cultures were transferred weekly by trypsinization and incubated at 37 °C in a humidified incubator containing 5% CO_2_ [12].

### 2.2. Preparation of Blueberry Extracts and Selection of Single Compounds

Blueberries from four different varieties were kindly received from the Dutch Blueberry Collective (Venlo, The Netherlands). The varieties were selected based on the level of antioxidant capacity as measured by oxygen radical absorbance capacity (ORAC) [13]. Two of these varieties, i.e., Draper and Bluecrop, have relatively low ORAC values, compared to the other two investigated varieties, i.e., Aurora and Elliot, which score relatively high in the ORAC assay. Blueberries were harvested and immediately frozen at −20 °C. Next, frozen blueberries were crushed into a powder using a stainless steel mortar which was placed in liquid nitrogen. Subsequently, blueberry powder was homogenized and dissolved in (end concentrations) 70% methanol/0.1% formic acid (Sigma-Aldrich, Zwijndrecht, The Netherlands). To remove undissolved blueberry material, tubes were centrifuged for 5 min at 3000 rpm. The supernatant, containing the blueberry extract was transferred to Eppendorf tubes and concentrated 10x using nitrogen gas, and stored at −80 °C until use.

In addition to the four different blueberry varieties, an extract was made of a blueberry–apple juice which was used in a previously performed human dietary intervention study investigating the chemopreventive properties of this blueberry–apple juice (BBAJ) [9,10,11]. Blueberry–apple juice was dissolved in in 70% methanol/0.1% formic acid (end concentrations), concentrated 10× using nitrogen gas, and stored at −80 °C until use.

Finally, 4 single compounds were selected based on their presence in the blueberry–apple juice and the blueberry varieties, i.e., vitamin C, quercetin 3-β-d-glucoside (Sigma Aldrich, Zwijndrecht, The Netherlands; abbreviated to quercetin), cyanidin-3-*O*-glucoside (cyanidin), and peonidin-3-*O*-glucoside (Extrasynthese, Lyon, France; peonidin) [9]. Vitamin C, cyanidin, and peonidin were dissolved in 1× phosphate buffered saline (PBS) (Life Technologies, Leusden, The Netherlands), and quercetin in dimethylsulfoxide (DMSO) (Sigma-Aldrich, Zwijndrecht, The Netherlands).

Characterization of the different extracts was carried out by Plant Research International (Wageningen UR, The Netherlands) using standard HPLC analysis (Table 1) as described before [14]. In the different extracts, the concentrations of the 4 different single compounds were measured.

### 2.3. Treatment and Isolation of Caco-2 Cells

For cytotoxicity assay, alkaline comet assay and gene expression analyses, twenty-four hours prior to the start of the experiments, the cells were trypsinized and transferred to 6-wellsplates at 80% confluence. Cells for electron spin resonance (ESR) spectroscopy measurements were cultured in T75 flasks. Treatment and isolation of cells for ESR measurements are described separately in the paragraph ‘ESR measurements’. 

**Figure 1:**
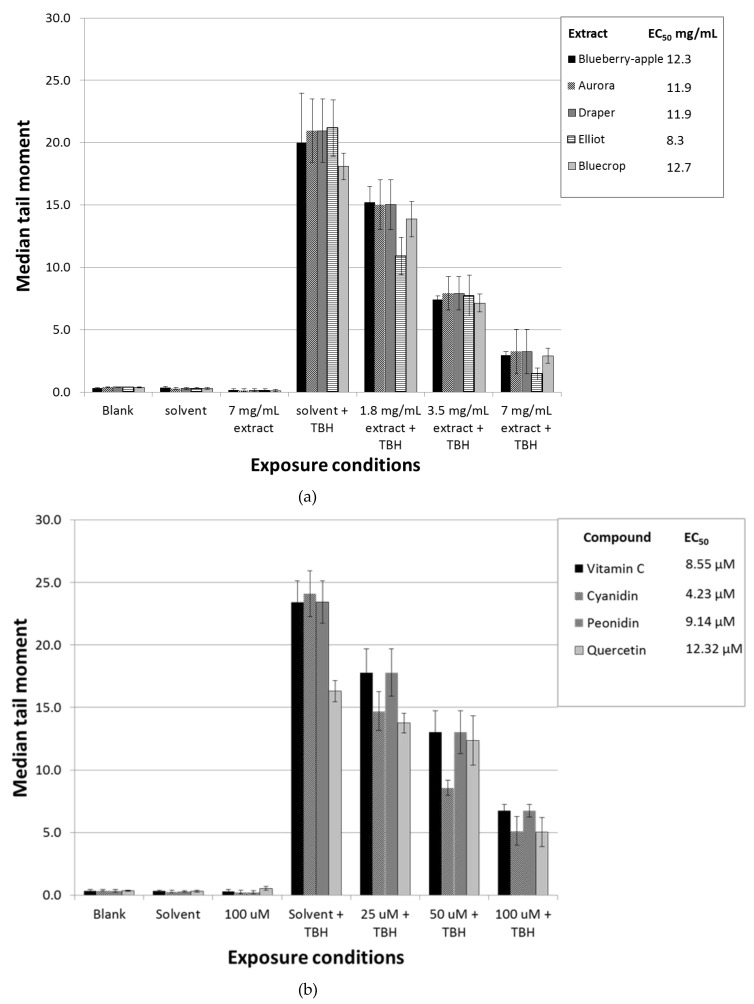
Level of DNA damage in Caco-2 cells as measured by the alkaline comet assay, expressed as average median tail moments. Error bars indicate standard deviations. Caco-2 cells were pre-incubated for 2 h with different concentrations of the extract of blueberry–apple juice or extracts of four different blueberry varieties (**a**), or single compounds (**b**) and subsequently exposed to 100 µM tert-butylhydroperoxide (TBH) for 1 h. Next, DNA strand breaks were measured using the alkaline comet assay. Pre-incubation for 2 h with medium, solvent control (0.5% end concentration of 70% methanol/0.1% formic acid), the highest concentration of the different extracts (i.e., 7 mg/mL), or 100 µM of single compounds did not induce any DNA damage. A dose-dependent decrease in DNA damage was observed for all blueberry extracts and single compounds tested (ANOVA, *P* < 0.01). In order to investigate which of the blueberry extracts and single compounds possessed the highest chemopreventive properties, linear log regression was applied. From the log linear regression equation, the EC_50_ was estimated which is shown in the legend.

**Figure 2:**
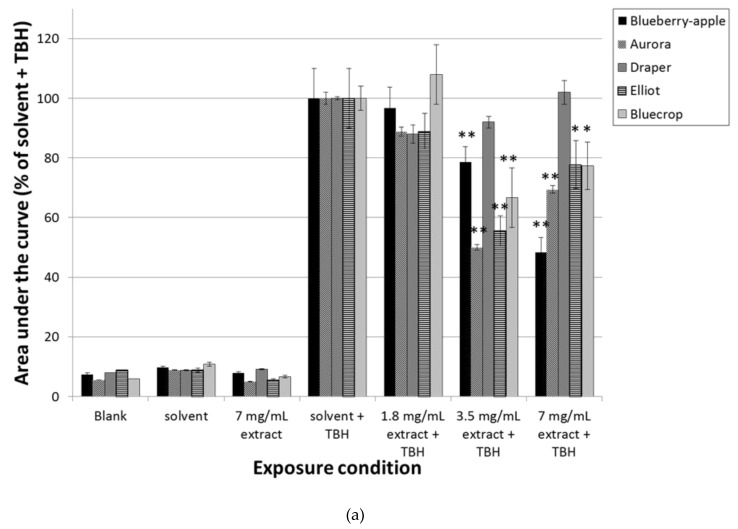

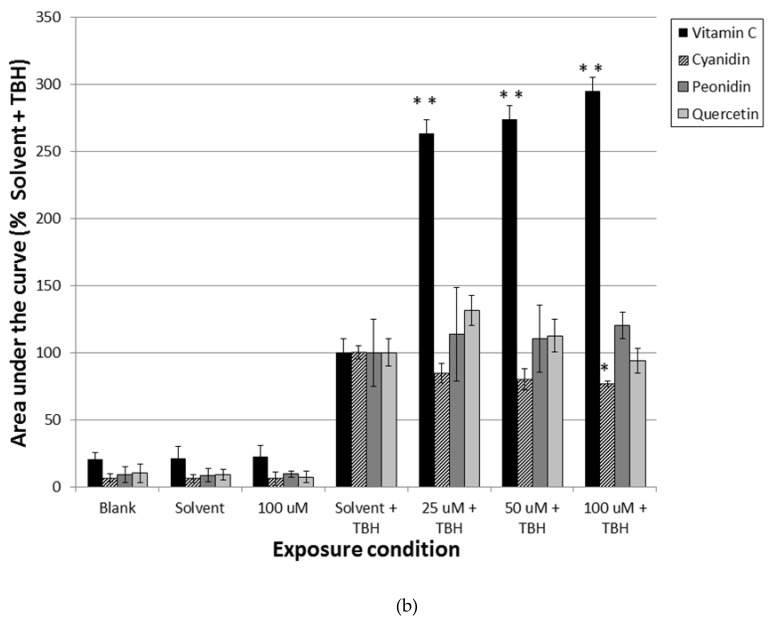
Radical formation in Caco-2 cells as measured by ESR spectroscopy. Results are expressed as percentage of solvent control levels. AUC: area under the curve of radical specific signals. Error bars indicate standard deviations. Caco-2 cells were pre-incubated for 2 h with different concentrations of the extract of blueberry–apple juice or extracts of four different blueberry varieties (**a**), or single compounds (**b**) and subsequently exposed to 150 µM tert-butylhydroperoxide (TBH) for 30 min. Pre-incubation for 2 h with medium, solvent control (0.5% end concentration of 70% methanol/0.1% formic acid), the maximal concentration of the different extracts (i.e., 7 mg/mL), or 100 µM of single compounds did not induce significant levels of radical formation. ** *P* < 0.01; * *P* < 0.05, significantly different from Caco-2 cells exposed to solvent control for 2 h and challenged with 150 µM TBH for 30 min.

Cells were pre-incubated with the different blueberry extracts or single compounds for different time points and at different concentrations. The selected concentrations did not result in significantly increased cell death (viability > 95%, File S1), induction of oxidative DNA damage (Figure 1A,B), or radical formation (Figure 2A,B). The highest concentration of the extracts that the Caco-2 cells were to be exposed to was determined by the maximal concentration of the solvent 70% methanol/0.1% formic acid in medium, which was 0.5% in all experiments. In addition to this highest concentration of blueberry extract (i.e., 7 mg/mL), Caco-2 cells were exposed to half of this concentration (3.5 mg/mL), and to a fourth (1.8 mg/mL). In order to maintain equal levels of methanol/formic acid, different concentrations of the extracts were prepared using serial dilutions with 70% methanol/0.1% formic acid. Caco-2 cells were pre-incubated with the different blueberry extract, the solvent, or medium for 2 and 24 h.

The 4 single compounds were tested in a concentration range of 0, 25, 50, and 100 µM and pre-incubated for 2, 6, 24, and 48 h. The final concentration of the solvent in the medium was 0.5%.

After pre-incubation, a subset of cells was challenged with the oxidant tert-butylhydroperoxide (TBH) (Sigma Aldrich, Zwijndrecht, The Netherlands). For Comet assay experiments, Caco-2 cells were challenged with 100 µM TBH for 1 h, as this exposure condition resulted in cell viability levels > 80%, and a moderate increase in oxidative DNA damage (Figure S1). The optimal exposure condition of Caco-2 cells in the ESR spectroscopy measurements was determined at 150 µm TBH for 30 min as at this condition cell viability levels were >80% and a significant increase in free radical formation was observed. Experiments were carried out in triplicate (Figure S2). After exposure, cells were washed twice with 1 mL Hank’s Balanced Salt Solution, without Ca and Mg (HBSS, Life Technologies, Leusden, The Netherlands), isolated by trypsinization, resuspended in 1× PBS and subsequently placed on ice. For gene expression experiments, cells were lysed in the culture dish using TRIzol^®^ Reagent (Invitrogen, Breda, The Netherlands), and stored at −20 °C until use. 

### 2.4. Cytotoxicity Assay

Cytotoxicity of the blueberry extracts, the single compounds, and TBH was measured using the trypan blue exclusion assay. Fifteen µL cell suspension was mixed with 15 µL 0.4% trypan blue solution (Life Technologies, Leusden, The Netherlands) and incubated for 1 min at 37 °C. The mixture was transferred to a Bürker counting chamber (Sigma Aldrich, Zwijndrecht, The Netherlands). The number of viable colorless cells and the number of dead blue cells were counted, and viability was calculated as percentage viable cells.

### 2.5. Alkaline Single-Cell Gel Electrophoresis (Comet Assay)

To establish the preventive effect of phytochemicals on the induction of genetic damage, the alkaline comet assay was used [15,16], according to the latest guidelines [16]. In order to detect single and double strand breaks as well as abasic sites where DNA repair is taking place Caco-2 cells were pre-incubated with or without the different blueberry extracts or one of the single compounds and subsequently challenged with the oxidant TBH. Cells were suspended in 1× PBS at a final cell concentration of 1 × 10^6^ cells/mL. Twenty-five microliter cell suspension was mixed with 75 µL low melting point agarose (Sigma Aldrich, Zwijndrecht, The Netherlands) and positioned on a 1.5% pre-coated agarose microscope slide. Further procedures were described earlier [15]. Comets were visualized using a Zeiss Axioskop fluorescence microscope. Randomly, 50 comets were analyzed using the Comet assay III software (Perceptive Instruments, Haverhill, UK). DNA damage was assessed as tail moment (TM, which is the product of tail DNA content and mean tail migration distance). Three slides were obtained from 1 well of cell culture plate and each experiment was performed in triplicate. Median values of tail moment and their standard deviations were calculated for further data analysis [12].

### 2.6. Electron Spin Resonance Spectroscopy Measurements

Caco-2 cells cultured in T75 culture flasks were washed with HBSS, isolated by trypsinization and suspended in 1 mL HBSS with or without the addition of different concentrations of blueberry extracts or individual phytochemicals, or solvent control, and incubated for 2 h in a CO_2_ incubator at 37 °C. Thirty minutes prior to the end of the pre-incubation time, the spin trapping agent α-(4-pyridyl-1-oxide)-*N*-tert-butylnitrone (POBN) (Sigma Aldrich, Zwijndrecht, The Netherlands) was added to the suspension at a final concentration of 50 mM, and incubated for 30 min in a 5% CO_2_ incubator at 37 °C, in order to detect the formation of hydroxyl radicals [17]. Next, cells were challenged with 150 µM TBH for 30 min. For each sample, a 100 µL glass capillary (Brand, Wertheim, Germany) was filled with homogenized cell suspension and sealed. After sealing, the capillary was immediately placed in the resonator of the ESR spectrometer. ESR spectra were recorded at room temperature on a Bruker EMX 1273 spectrometer equipped with an ER 4119HS high sensitivity cavity and 12 kW power supply operating at X band frequencies. The modulation frequency of the spectrometer was 100 kHz. Instrumental conditions for the recorded spectra were as follows: magnetic field, 3490 G; scan range, 60 G; modulation amplitude, 1 G; receiver gain, 1 × 10^5^; microwave frequency, 9.85 GHz; power, 50 mW; time constant, 40.96 ms; scan time, 20.97 s; number of scans, 35. Spectra were quantified by peak surface measurements through double integration of the ESR spectrum using the WIN-EPR spectrum manipulation program (Bruker BioSpin, Wormer, The Netherlands). All experiments were performed in triplicate [18]. 

### 2.7. Real-Time qPCR

Caco-2 cells were treated with either each extract at a maximal concentration of 7 mg/mL for 2 or 24 h or with 50 µM of one of the four single compounds for 2, 6, 24, or 48 h. Cells were lysed in the culture dish using 200 µL TRIzol^®^ Reagent (Invitrogen, Breda, The Netherlands), and stored at −20 °C until RNA isolation. RNA isolation was performed according to the manufacturer’s protocol followed by a cleanup using the RNeasy Mini Kit (Qiagen, Venlo, The Netherlands) with DNase treatment. RNA quantity and purity were inspected by measuring their absorbance at 260 and 280 nm. Furthermore, quality of RNA was assessed by automated gel electrophoresis on an Agilent 2100 Bioanalyzer (Agilent Technologies, Amstelveen, The Netherlands). All samples held an RNA integrity number of >8. 

The iScript^TM^ cDNA synthesis kit (Bio-Rad, Veenendaal, The Netherlands) was used to synthesize cDNA, according to the manufacturer’s instructions. Five hundred ng of RNA was applied for each reaction together with 4 µL × iScript reaction mixture and 1 µL iScript reverse transcriptase. Nuclease free water was added to constitute the final volume of 20 μl. Amplified cDNA products were diluted 10× with 180 µL of nuclease free water and used for real-time RT-PCR. Real-time RT-PCR was performed in a final volume of 25 µL on a MyiQ single color RT-PCR detection system (Bio-Rad Veenendaal, The Netherlands). Each RT-PCR tube contained 12.5 µL 2× SYBR GREEN supermix, 2.5 µL 3 µM of forward and reverse primer, 2.5 µL nuclease free water and 5 µL diluted cDNA. 

Genes of interest were chosen based on the results of the previously performed human dietary intervention study (HDIS) in which volunteers consumed 1 L of a blueberry–apple juice per day for four weeks in order to investigate the chemopreventive properties of the fruit juice rich in phytochemicals [9,11]. A whole genome gene expression analysis was performed on total RNA isolated from lymphocytes which were isolated before and after the intervention. Pathway analysis of the significantly modulated genes showed strong but complex gene expression changes in pathways signaling for apoptosis, immune response, cell adhesion, and lipid metabolism [9,10]. These pathways indicate increased apoptosis, upgraded growth control, induced immunity, reduced platelet aggregation and activation, blood glucose homeostasis, and regulation of fatty acid metabolism. A selection of 18 genes was made based on biological relevance and presence in these pathways, and includes v-rel reticuloendotheliosis viral oncogene homolog B (*RelB*), interleukin-8 (*IL8*), B-cell CLL/lymphoma 2 (*BCL2*), myeloid cell leukemia sequence 1 (BCL2-related) (*MCL1*), Catenin (Cadherin-Associated Protein), beta 1 (*CTNNB1*), caspase 8 (*CASP8*), *CAPS3*, *CASP10*, phosphoinositide-3-kinase, catalytic (p110 alpha) polypeptide (*PIK3CA*), phosphoinositide-3-kinase, regulatory subunit 1 (p85 alpha) (*PIK3R1*), v-akt murine thymoma viral oncogene homolog 2 (AKT2), signal transducer and activator of transcription 1 (*STAT1*), *STAT3*, *STAT6*, janus kinase 1 (*JAK1*), *JAK2*, tyrosine kinase 2 (*TYK2*), and hypoxia inducible factor 1, alpha subunit (*HIF1A*) (see Table S1 for involved biological pathways, direction of gene expression effect in the human dietary intervention study, and forward and reverse primer sequence). The expression levels of target genes were compared with that of beta-actin (ACTB) as a housekeeping gene. 

### 2.8. Hierarchical Clustering Analyses

Hierarchical clustering analysis was used to cluster the different treatments based on gene expression results (http://heatmapper.ca). Hierarchical clustering is an algorithm that groups similar objects into groups called clusters. The endpoint is a set of clusters, where each cluster is distinct from each other cluster, and the objects within each cluster are broadly similar to each other [19]. The main output of the hierarchical clustering is a dendrogram, which shows the hierarchical relationship between the clusters. As clustering method complete linkage was used, and distance between the clusters was computed based on Spearman rank correlation, the dendogram is displayed as a tree diagram, in which the conditions are the different leaves and the branches show the leaves which are most similar to each other. The different conditions are displayed on the y-axis and the genes are shown on the x-axis.

### 2.9. Statistical Analyses

Data are presented as median tail moment ± SD (alkaline comet assay), or mean area under the curve (AUC) ± SD (ESR measurements) as a percentage compared with the solvent control exposed to TBH. Values were analyzed by one-way analysis of variance (ANOVA) followed by *t*-test to assess the protective effect of each extract and single compounds. A *P*-value of <0.05 was considered statistically significant.

To evaluate different protective potentials among different extracts or single compounds, linear regression analysis of tail moment versus log concentration of the extracts or single compounds was adapted on the basis of the statistical methods used by Noroozi et al. (1998) [20]. The effective concentration (EC_50_) was calculated based on the concentration that would decrease the tail moment by 50% in the presence of the extracts or single compounds. Extrapolation was used to estimate EC_50_. 

## 3. Results

### 3.1. Pre-Incubation with Blueberry Extracts and Single Compounds Protect Caco-2 Cells against Oxidative DNA Damage

To examine the chemopreventive properties of the different blueberry extracts and the single compounds, Caco-2 cells were pre-incubated for 2 h with different concentrations and subsequently exposed to 100 µM TBH for 1 h. A dose-dependent decrease in DNA strand breaks was observed for all blueberry extracts and phytochemicals tested (*P* < 0.01) (Figure 1A,B, respectively). In order to investigate which of the blueberry extracts and single compounds possessed the highest chemopreventive properties, linear log regression was applied. From the log linear regression equation, the EC_50_ was estimated (Figure 1A). The extract of the blueberry variety Elliot showed the highest potential to reduce DNA strand breaks (EC_50_ = 8.3 mg/mL), followed by Aurora and Draper (EC_50_ = 11.9 mg/mL). The extract of Bluecrop (EC_50_ =12.7 mg/mL) showed the least potential to decrease the number of DNA strand breaks, compared to the other varieties. The extract generated from the blueberry–apple juice performed only slightly better compared to the extract of Bluecrop (EC_50_ = 12.3 mg/mL). From the single compounds, cyanidin showed the highest chemopreventive properties (EC_50_ = 4.23 µM), followed by vitamin C (EC_50_ = 8.55 µM), and peonidin (EC_50_ = 9.14 µM). The concentration of quercetin required to reduce the number of strand breaks by 50% was almost four times higher than cyanidin (EC_50_ = 12.32 µM), and showed the least chemopreventive properties. 

### 3.2. Pre-Incubation with Blueberry Extracts and Individual Phytochemicals Reduces Free Radical Generation in Caco-2 Cells

In order to get more insight into the chemopreventive mechanisms of the different blueberry extract and single compounds, Caco-2 cells were also pre-incubated for 2 h with different concentrations, and subsequently challenged for 30 min with 150 µM TBH after which free radical formation was measured by ESR spectroscopy. The extract of blueberry–apple juice was able to dose-dependently decrease the formation of free radicals (*P* < 0.01) (Figure 2A,B). In addition, the extracts of Elliot, Aurora, and Bluecrop were able to reduce the generation of free radicals, but only at a concentration of 3.5 mg/mL and 7 mg/mL (*P* < 0.01). Draper was not able to diminish the amount of free radicals after pre-incubation and subsequent exposure to TBH.

The effects of the single compounds on reduction of free radical formation, was less pronounced. Only exposure to cyanidin at the highest concentration showed a decrease of almost 25% of free radical formation after TBH exposure (*P* < 0.05). On the contrary, pre-incubation with vitamin C resulted in a large increase of radical formation after challenge with TBH (*P* < 0.01).

### 3.3. Modulation of Gene Expression by the Blueberry Extracts and Individual Phytochemicals

The relative mRNA levels of a selection of 18 genes measured in the human intervention with BBAJ were also evaluated here to examine the effects of the different extracts and individual compounds on gene expression and chemoprevention. The column charts for each gene which shows the relative level of mRNA for each blueberry extract and each single compound, as compared to the house keeping gene, beta-actin, can be found in File S1. In Table S2, the direction of modulation is shown for each gene after exposure to the different blueberry extracts and single compounds at each tested concentration and exposure time. Furthermore, it provides an overview of the number of genes which are equally modulated after each exposure time by the different blueberry extracts or single compounds as compared to the gene expression changes induced by the blueberry–apple juice in the large scale human dietary intervention study. Exposure to a relatively shorter duration of 2 and 6 h, reveal the lowest number of similarly modulated genes for both the extracts and the single compounds (no. of genes for the extracts <39%; and for the single compounds <28%). The gene expression effects which are induced by the extract of the blueberry–apple juice, as well as by the different blueberry extracts show a large overlap with the gene expression effects in the dietary intervention study, at the longest incubation time of 24 h. The extract of the blueberry variety Elliot is able to induce for almost 80% of the genes similar effects (78%; 14 genes out of 18 genes are modulated in the same direction), and performs even better as compared to the results of the extract of the blueberry–apple juice (61%; 11 genes out of 18 genes are modulated in the same direction). The extract of the blueberry variety Draper shows the lowest number of similar gene expression effects, but still 44% of the genes are modulated in a comparable way (8 genes out of 18 genes). On the other hand, the single compounds are overall not able to induce many equal effects on gene expression level, at all time points. In this respect, incubation for 48 h with quercetin results in the highest number of similarly modulated genes (44%), while vitamin C and cyanidin can do this for only 17% of the genes.

**Figure 3:**
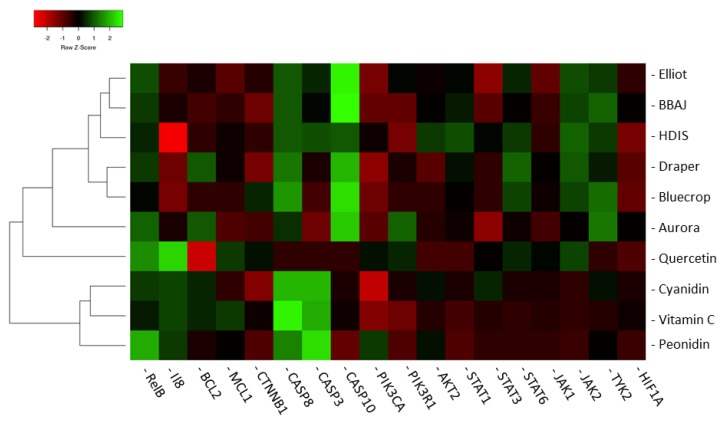
Hierarchical clustering dendrogram of different blueberry extracts (Elliot, Draper, Bluecrop, and Aurora), the extract of the blueberry–apple juice (BBAJ) from the human dietary intervention, and of four single compounds (quercetin, vitamin C, peondin, and cyanidin) based on the effect on the expression of 18 genes selected from a previously conducted human dietary interventions study. In order to compare these gene expression profiles with the profile generated in the human dietary intervention study, the gene expression profile of the human dietary intervention study is included as well (HDIS). Hierarchical clustering analysis was carried out using http://heatmapper.ca. As clustering method complete linkage was used, and distance between the clusters was computed based on Spearman rank correlation. The different conditions are displayed on the y-axis and the genes are shown on the x-axis.

Hierarchical clustering analysis was used to examine the similarities between all groups (at the longest time point of exposure), i.e., the different extracts, the single compounds, and the effect of the human dietary intervention study (Figure 3). The different gene expression profiles are compared among all groups and not solely to the profile of the human dietary intervention study. Gene expression profiles which are most similar to each other are gene expression profiles induced by Elliot and the extract of the blueberry–apple juice, by Draper and Bluecrop, and by cyanidin and vitamin C. The gene expression profile of the human dietary intervention study is most similar to the cluster of Elliot and the extract of blueberry–apple juice tested in vitro. Aurora induced a gene expression profile which is least comparable to the gene expression profiles generated by the different blueberry extracts and the effect in the human dietary intervention study. The gene expression profile induced by quercetin is more similar to the gene expression profiles induced by the blueberry extracts and the juice of human dietary intervention study, as compared to the other single compounds vitamin C, peonidin, and cyanidin.

## 4. Discussion

In this study we evaluated the effect of different blueberry extracts with regard to their antioxidant capacity and their potency to protect cultured human colonocytes against DNA damage. These findings were compared to the effects of single compounds that are known contributors to the antioxidant value of blueberries and present in different amounts in different blueberry varieties. Overall these results were used to validate the effects established in a previous conducted human dietary intervention study evaluating the antioxidant effects of a blueberry–apple juice. The underlying hypothesis is that complex combinations of bioactive phytochemicals as present in whole fruits are more efficient in inducing antioxidant effects as compared to single compounds in view of potentially synergistic effects as a consequence of simultaneously induced molecular responses [4].

We found that extracts of all different blueberry varieties and of the blueberry–apple juice from the human dietary intervention study, used in our study are capable of reducing levels of DNA damage induced by TBH. Extracts of Elliot, Aurora, Bluecrop, and blueberry–apple juice were also able to reduce the generation of free radicals, but only at the higher concentrations (3.5 mg/mL and 7 mg/mL) (*P* < 0.01), whereas extracts of Draper were not able to diminish the amount of free radicals formed. Elliot, the variant with the highest ORAC value, shows the highest capacity to prevent DNA damage (EC_50_ of 8.3 mg/mL). Of the single compounds, cyanidin showed the highest chemopreventive properties (EC_50_ = 4.23 µM), whereas quercetin was found to be the least effective in reducing the number of strand breaks. Cyanidin was the only single compound showing a significantly decreased level of free radical formation at the highest concentration. This polyphenol is considered the widest spread anthocyanin present in fruits and vegetables and it is known to be a strong natural antioxidant, stronger than several other antioxidants such as resveratrol and vitamin C [21]. Also Petruk et al. (2017) reported that cyanidin and malvidin derivatives are the most active molecules from Acai berries able to counteract the negative effects induced by oxidative stress [22]. The observation that single compounds are much less effective in radical scavenging as compared to the blueberry extracts supports the relevance of combinatory effects. Furthermore, although the reduction of oxidative DNA damage for both the individual compounds and the blueberry extracts is similar at the chosen concentration and dilution ranges, the tested concentrations of the individual compounds are much higher than their natural concentrations in the blueberry extracts (Table 1), which substantiates the importance of combinatory effects even further. The remarkable observation that vitamin C increases radical measurements is the consequence of its known capacity to stimulate redox cycling and simultaneously scavenge oxygen radicals to form a stable vitamin C radical, a process that has been described previously [23].

The finding that blueberry extracts reduce DNA damage to a higher extent than ROS formation, suggests the involvement of other mechanisms than radical-mediated processes in the protection against oxidative cell damage. In our human intervention study with blueberry–apple juice, we observed indeed a strong gene expression response reflecting a wide range of biological processes modulated by the intervention [9,10]. Among the identified pathways in association with reduced oxidative stress, we found increased apoptosis, induced immunity, upgraded growth control, and regulation of fatty acid metabolism, all processes that may be of relevance in the development of chronic diseases. Using a panel of 18 genes selected from this study, we evaluated the ability of different blueberry extracts including the extract of the blueberry–apple juice from the in vivo study, and single compounds to induce gene expression profiles in caco-2 cells that are in line with those observed in vivo. Although different types of blueberries have been compared with regard to their antioxidant potential [24] differences in their capacity to modulate gene expression of relevant genes in chronic diseases have never been evaluated before. Khoo et al. (2017) recently reviewed the biological effect of anthocyanins and reported different mechanisms and pathways involved in the protective effects, including free-radical scavenging pathway, cyclooxygenase pathway, apoptosis, mitogen-activated protein kinase pathway, and inflammatory cytokine signaling. Although the genes in our selected profile are not completely identical, the processes in which they are involved are clearly overlapping, including inflammation and apoptosis [25].

In the comparison of gene expression responses after exposure of Caco-2 cells to different blueberry extracts, we found a large resemblance with the gene expression profile induced by the blueberry–apple juice that was previously applied in our human dietary intervention study. At the latest time point of exposure (24 h), the extract of the Elliot blueberry was capable of inducing almost 80% of genes in the same direction. Also the hierarchical clustering analyses showed that the gene expression profile induced by Elliot showed the largest resemblance to the profile induced by the blueberry–apple juice, both in vitro and in vivo. This suggests that consumption of this blueberry variety may result in comparable molecular responses and health effects as observed in the intervention study. Furthermore, the gene expression profile of the extract of the blueberry–apple juice also shows strong similarities with the profile obtained in humans during an intervention study [9,10], thereby validating the gene expression profile established in vivo in an in vitro cell model. On the other hand, the effects induced by the Draper extract only showed 44% resemblance with the in vivo study. This is also the blueberry variety with the lowest level of phytochemicals and which showed the lowest radical scavenging capacity. The single compounds showed much less similarity with the response induced by the blueberry extracts. This indicates that the single compounds are also less likely to induce the same molecular processes that were observed in the human study in relation to preventive health effects. Quercetin induces the most comparable gene expression response at all time points, which may be explained by the fact that the blueberry–apple juice of the intervention study was specifically designed to have high levels of quercetin. The resemblance of the gene expression responses at 48 h induced by cyanidin, vitamin C, and peonidin is much less, and these compounds are therefore less likely to induce the full biological response individually.

## 5. Conclusions

Overall, we conclude that different blueberry varieties show varying capacities to scavenge oxygen free radicals and to protect against DNA damage induced by oxidative stress. The variant with the highest ORAC value (Elliot) shows the strongest effects, and of the individual compounds cyanidin appears to have the best capacity to contribute to the antioxidant capacity.

Gene expression analyses in human studies have identified which molecular processes are responsible for the health benefits of blueberry derived phytochemicals. The nature of these processes, including immune responses, apoptosis, lipid metabolism, and platelet aggregation, indicates the relevance of the effects in relation to the reduction of oxidative stress and related diseases such as cardiovascular illnesses, cancer, and diabetes. Considering these molecular mechanisms, a causal relationship between consumption of blueberries and reported health impacts is made more plausible. Comparison of gene expression profiles induced by blueberry extracts and single compounds shows that more complex combinations of phytochemicals are more likely to induce biological responses that contribute to the reduction of disease risk and human health promotion. Finally, the results of this study provides a proof of concept that in vitro systems using gene expression changes can be used to characterize biological activity of different combinations of bioactive compounds and may be further developed in to a tool/instrument to rank food products in terms of potential health promoting effects. 

## Figures and Tables

**Table 1:** Concentrations of bioactive compounds in blueberry–apple juice extract, and in extracts of different blueberry varieties, dissolved in medium at maximal concentration of 0.5% of the solvent 70% methanol/0.1% formic acid (µM).

	Blueberry–Apple Juice	Aurora	Bluecrop	Draper	Elliot
Cyanidin-3-*O*-glucoside	0.6	4.7	1.4	2.7	4.7
Peonidin-3-*O*-glucoside	1.7	4.3	1.5	2.7	2.7
Quercetin-3-β-d-glucoside	6.9	1.1	1.2	1.2	1.1
Vitamin C	0.6	<0.5 *	<0.5 *	<0.5 *	<0.5 *

* Below limit of detection of 0.5 µM.

## Supplementary Materials

The following are available online at http://www.mdpi.com/2072-6643/11/1/61/s1, Figure S1: Level of DNA damage in Caco-2 cells as measured by the alkaline comet assay, expressed as average median tail moments; Figure S2: Radical formation in Caco-2 cells as measured by ESR spectroscopy. Table S1: Overview of genes for Real-Time qPCR analyses; Table S2: Comparison of gene expression effects in a previous performed large scale intervention study investigating blueberry apple juice [9,10], with the gene expression effects in Caco-2 cells pre-incubated with extract of blueberry–apple juice, extracts of four different blueberry varieties, and four individual phytochemicals. File S1: Cytotoxicity results of incubations with all tested concentrations of blueberry–apple juice, extracts of four different blueberry varieties, and four individual phytochemicals; RT-PCR results of incubations with highest concentration of extracts of blueberry–apple juice (BBAJ), Bluecrop, Draper, Elliot, or Aurora; RT-PCR results of incubations with 50 uM vitamin C, quercetin, peonidin, or cyanidin.

## References

[1] World Cancer Research Fund, American Institute for Cancer Research (2007). Nutrition, Physical Activity, and the Prevention of Cancer: A Global Perspective.

[2] De Kok T.M., van Breda S.G., Manson M.M. (2008). Mechanisms of combined action of different chemopreventive dietary compounds: A review. Eur. J. Nutr..

[3] Kok T.M., Breda S.G., Briede J.J. (2012). Genomics-based identification of molecular mechanisms behind the cancer preventive action of phytochemicals: Potential and challenges. Curr. Pharm. Biotechnol..

[4] Van Breda S.G.J., de Kok T. (2017). Smart Combinations of Bioactive Compounds in Fruits and Vegetables May Guide New Strategies for Personalized Prevention of Chronic Diseases. Mol. Nutr. Food Res..

[5] Van Breda S.G., van Agen E., Engels L.G., Moonen E.J., Kleinjans J.C., van Delft J.H. (2004). Altered vegetable intake affects pivotal carcinogenesis pathways in colon mucosa from adenoma patients and controls. Carcinogenesis.

[6] Grainger E.M., Schwartz S.J., Wang S., Unlu N.Z., Boileau T.W., Ferketich A.K., Monk J.P., Gong M.C., Bahnson R.R., DeGroff V.L. (2008). A combination of tomato and soy products for men with recurring prostate cancer and rising prostate specific antigen. Nutr. Cancer.

[7] Thompson H.J., Heimendinger J., Diker A., O’Neill C., Haegele A., Meinecke B., Wolfe P., Sedlacek S., Zhu Z., Jiang W. (2006). Dietary botanical diversity affects the reduction of oxidative biomarkers in women due to high vegetable and fruit intake. J. Nutr..

[8] George T.W., Paterson E., Waroonphan S., Gordon M.H., Lovegrove J.A. (2012). Effects of chronic consumption of fruit and vegetable puree-based drinks on vasodilation, plasma oxidative stability and antioxidant status. J. Hum. Nutr. Diet..

[9] van Breda S.G., Wilms L.C., Gaj S., Jennen D.G., Briede J.J., Helsper J.P., Kleinjans J.C., de Kok T.M. (2014). Can transcriptomics provide insight into the chemopreventive mechanisms of complex mixtures of phytochemicals in humans?. Antioxid. Redox Signal..

[10] Van Breda S.G., Wilms L.C., Gaj S., Jennen D.G., Briede J.J., Kleinjans J.C., de Kok T.M. (2015). The exposome concept in a human nutrigenomics study: Evaluating the impact of exposure to a complex mixture of phytochemicals using transcriptomics signatures. Mutagenesis.

[11] Wilms L.C., Boots A.W., de Boer V.C., Maas L.M., Pachen D.M., Gottschalk R.W., Ketelslegers H.B., Godschalk R.W., Haenen G.R., van Schooten F.J. (2007). Impact of multiple genetic polymorphisms on effects of a 4-week blueberry juice intervention on ex vivo induced lymphocytic DNA damage in human volunteers. Carcinogenesis.

[12] Hebels D.G., Jennen D.G., Kleinjans J.C., de Kok T.M. (2009). Molecular signatures of N-nitroso compounds in Caco-2 cells: Implications for colon carcinogenesis. Toxicol. Sci..

[13] Helsper J.P. (2008). Antioxidantcapaciteit en Gehalten aan Anthocyanen en Fenolische Verbindingen in in Nederland Geteelde Rassen van Blauwe bes (Vaccinium corymbosum L.).

[14] Linskens H.-F., Jackson J.F. (1987). High Performance Liquid Chromatography in Plant Sciences.

[15] Singh N.P., McCoy M.T., Tice R.R., Schneider E.L. (1988). A simple technique for quantitation of low levels of DNA damage in individual cells. Exp. Cell Res..

[16] Tice R.R., Agurell E., Anderson D., Burlinson B., Hartmann A., Kobayashi H., Miyamae Y., Rojas E., Ryu J.C., Sasaki Y.F. (2000). Single cell gel/comet assay: Guidelines for in vitro and in vivo genetic toxicology testing. Environ. Mol. Mutagen..

[17] Tiku M.L., Yan Y.P., Chen K.Y. (1998). Hydroxyl radical formation in chondrocytes and cartilage as detected by electron paramagnetic resonance spectroscopy using spin trapping reagents. Free Radic. Res..

[18] Hebels D.G., Briede J.J., Khampang R., Kleinjans J.C., de Kok T.M. (2010). Radical mechanisms in nitrosamine- and nitrosamide-induced whole-genome gene expression modulations in Caco-2 cells. Toxicol. Sci..

[19] Kimes P.K., Liu Y., Neil Hayes D., Marron J.S. (2017). Statistical significance for hierarchical clustering. Biometrics.

[20] Noroozi M., Angerson W.J., Lean M.E. (1998). Effects of flavonoids and vitamin C on oxidative DNA damage to human lymphocytes. Am. J. Clin. Nutr..

[21] Amorini A.M., Fazzina G., Lazzarino G., Tavazzi B., Di Pierro D., Santucci R., Sinibaldi F., Galvano F., Galvano G. (2001). Activity and mechanism of the antioxidant properties of cyanidin-3-*O*-β-glucopyranoside. Free Radic. Res..

[22] Petruk G., Illiano A., Del Giudice R., Raiola A., Amoresano A., Rigano M.M., Piccoli R., Monti D.M. (2017). Malvidin and cyanidin derivatives from acai fruit (Euterpe oleracea Mart.) counteract UV-A-induced oxidative stress in immortalized fibroblasts. J. Photochem. Photobiol. B Boil..

[23] Briede J.J., De Kok T.M., Hogervorst J.G., Moonen E.J., Op Den Camp C.L., Kleinjanst J.C. (2005). Development and application of an electron spin resonance spectrometry method for the determination of oxygen free radical formation by particulate matter. Environ. Sci. Technol..

[24] Drozdz P., Seziene V., Pyrzynska K. (2017). Phytochemical Properties and Antioxidant Activities of Extracts from Wild Blueberries and Lingonberries. Plant Foods Hum. Nutr..

[25] Khoo H.E., Azlan A., Tang S.T., Lim S.M. (2017). Anthocyanidins and anthocyanins: Colored pigments as food, pharmaceutical ingredients, and the potential health benefits. Food Nutr. Res..

