# Supplementary material for: Improved Preventive Effects of Combined Bioactive Compounds Present in Different Blueberry Varieties as Compared to Single Phytochemicals
Source: Nutrients. 2018 Dec 29;11(1):61. doi: 10.3390/nu11010061 (PMC6356906; doi:10.3390/nu11010061)
Supplement: Supplementary file 1 [file nutrients-11-00061-s001.zip › Supplementary data Table 1.docx]

**Supplementary data Table 1. Overview of genes for Real-Time qPCR analyses**

| Gene ID | Direction of gene expression effect^1^ | Involved biological pathway | Forward primer | Reverse primer |
| --- | --- | --- | --- | --- |
| RelB | ↑ | Immune response | TCCCAACCAGGATGTCTAGC | AGCCATGTCCCTTTTCCTCT |
| IL8 | ↓ | Immune response | CTTGGCAGCCTTCCTGATTT | TTCTTTAGCACTCCTTGGCAAAA |
| BCL2 | ↓ | Apoptosis | GATTGTGGCCTTCTTTGAG | CAAACTGAGCAGAGTCTTC |
| MCL1 | ↓ | Apoptosis | AAGAATTCATGTTTGGCCTC | AAGAATTCCTATCTTATTAGA |
| CTNNB1 | ↓ | Cell adhesion | GAAAATCCAGCGTGGACAATG | GGACAGTATGCAATGACTCG |
| CASP8 | ↑ | Apoptosis | AGAGTCTGTGCCCAAATCAAC | GCTGCTTCTCTCTTTGCTGAA |
| CASP3 | ↑ | Apoptosis | CTCGGTCTGGTACAGATGTCGA | CATGGCTCAGAAGCACACAAAC |
| PIK3CA | ↓ | Immune response  Cell adhesion  Lipid metabolism | GACGACTTTGTGACCTTCG | GAAGTCCTGTACTTCTGGAT |
| CASP10 | ↑ | Apoptosis | ACAAGGAAGCCGAGTCGTATCA | TGGTTCCGATTCATCCTGTACA |
| PIK3R1 | ↓ | Immune response  Cell adhesion  Lipid metabolism | AACCGAAACAAAGCGGAGAA | TTGACTTCGCCGTCTACCACT |
| AKT2 | ↑ | Immune response  Lipid metabolism | CAGCTGGGAGACCCAAGA | CACACGCTGTCACCTAGCTT |
| STAT1 | ↑ | Immune response | CTAGTGGAGTGGAAGCGGAG | CACCACAAACGAGCTCTGAA |
| STAT3 | ↓ | Immune response | CTTTGAGACCGAGGTGTATCACC | GGTCAGCATGTTGTACCACAGG |
| STAT6 | ↑ | Immune response | CTGGGGTGGTTTCCTCTTG | TGCCCGGTCTCACCTAACTA |
| JAK1 | ↓ | Immune response | GGAGTGCAGTATCTCTCCTCTCT | CCATGCCCAGGCACTCATTTTCA |
| JAK2 | ↑ | Immune response | GATTTCAGGCCTGCTTTCAG | ATGTTCCTTGTTGCCAGGTC |
| TYK2 | ↑ | Immune response | TGGCTTGGAAGATGGTGGTG | GTTCCGGCCACACACATTACC |
| HIF1A | ↓ | Immune response | GAAAGCGCAAGTCTTCAAAG | TGGGTAGGAGATGGAGATGC |
| ACTB | Housekeeping gene |  | AGAGCTACGAGCTGCCTGAC | AGCACTGTGTTGGCGTACAG |

1 Direction of gene expression effect in the previously performed large-scale human dietary intervention study investigating the chemopreventive effect of blueberry-apple juice (Wilms *et al.* 2007, van Breda *et al.* 2014, 2015).
