# Supplementary material for: Improved Preventive Effects of Combined Bioactive Compounds Present in Different Blueberry Varieties as Compared to Single Phytochemicals
Source: Nutrients. 2018 Dec 29;11(1):61. doi: 10.3390/nu11010061 (PMC6356906; doi:10.3390/nu11010061)
Supplement: Supplementary file 1 [file nutrients-11-00061-s001.zip › Supplementary data Table 2.docx]

**Supplementary data Table 2. Comparison of gene expression effects in a previous performed large scale intervention study investigating blueberry apple juice (**[**9**](#_ENREF_9)**) with the gene expression effects in Caco-2 cells pre-incubated with extract of blueberry-apple juice, extracts of four different blueberry varieties, and four individual phytochemicals**

| Gene ID | Direction of gene expression effect^1^ | Extracts^2,3^ | | | | | Individual phytochemicals^3,4^ | | | |
| --- | --- | --- | --- | --- | --- | --- | --- | --- | --- | --- |
|  |  | Blueberry-apple juice | Bluecrop | Draper | Elliot | Aurora | Vitamin C | Quercetin | Peonidin | Cyanidin |
| RelB | ↑ | X | X | T2 | T2  **T24** | T2  **T24** | T2  T6 | T2  **T48** | **T48** | X |
| IL8 | ↓ | **T2**  **T24** | **T2**  **T24** | **T2**  **T24** | **T2**  **T24** | **T2**  **T24** | **T2**  **T6** | **T2**  **T6**  T24  T48 | **T2**  T48 | X |
| BCL2 | ↓ | **T2**  **T24** | **T24** | X | **T24** | X | X | **T2**  **T48** | X | X |
| MCL1 | ↓ | T2  **T24** | T2  **T24** | **T24** | T2  **T24** | **T2**  **T24** | X | **T2**  T24  T48 | X | X |
| CTNNB1 | ↓ | **T24** | X | **T2**  **T24** | **T2**  **T24** | **T2**  **T24** | X | **T2**  T48 | **T2**  **T6**  **T24** | **T48** |
| CASP8 | ↑ | T2 | **T24** | X | T2  **T24** | T2 | **T48** | T2  **T24**  **T48** | **T48** | **T48** |
| CASP3 | ↑ | X | T24 | T2  T24 | T2 | T2  T24 | T2  T6  T24  **T48** | T2  **T24**  **T48** | T2  T6  **T48** | T2  T6  **T48** |
| PIK3CA | ↓ | **T24** | **T24** | **T2**  **T24** | **T24** | **T2**  **T24** | **T2**  **T24**  **T48** | **T2**  T6  **T24**  T48 | T2  T6  T24  T48 | **T48** |
| CASP10 | ↑ | **T24** | **T2**  **T24** | X | T2  **T24** | T2  **T24** | **T2** | **T6**  **T48** | T2  T24 | T24 |
| PIK3R1 | ↓ | **T2**  **T24** | **T24** | **T2**  **T24** | **T24** | **T2**  **T24** | T2  **T6**  **T48** | T48 | T2  T6  T24 | **T6** |
| AKT2 | ↑ | T24 | **T2**  T24 | T24 | X | T24 | T6  T24 | X | T2  **T24** | **T24** |
| STAT1 | ↑ | X | **T2** | T24 | X | T24 | **T24**  T48 | X | X | X |
| STAT3 | ↓ | **T24** | T2  **T24** | **T2**  **T24** | **T24** | **T2**  **T24** | T2 | T48 | T2  T6  T24 | X |
| STAT6 | ↑ | X | X | X | X | T2  T24 | **T24** | **T24**  **T48** | X | X |
| JAK1 | ↓ | T2  **T24** | T2  **T24** | **T2**  **T24** | **T24** | T2  **T24** | T2  T24 | T48 | T2  T6  T24 | X |
| JAK2 | ↑ | T2 | T2 | T2 | T2  **T24** | T2  T24 | T6 | **T6**  **T48** | X | T48 |
| TYK2 | ↑ | **T2**  **T24** | **T2**  **T24** | T24 | **T24** | **T24** | **T24** | **T24**  **T48** | X | X |
| HIF1A | ↓ | **T24** | **T24** | **T2**  **T24** | **T2**  **T24** | **T2**  **T24** | T24 | T24 | T2  T24 | **T48** |
| Number of genes modulated in the same direction as in the *in vivo* study (%) | | | | | | | | | | |
| T=2 for extracts and for single compounds | | **4** (22) | **4** (22) | **7** (39) | **3** (17) | **6** (33) | **3** (17) | **5** (28) | **2** (11) | **0** (0) |
| T=6 for single compounds | | NA | NA | NA | NA | NA | **2** (11) | **3** (17) | **1** (6) | **1** (6) |
| T=24 for extracts and for single compounds | | **11** (61) | **11** (61) | **8** (44) | **14** (78) | **11** (61) | **4** (22) | **5** (28) | **2** (11) | **1** (6) |
| T = 48 for single compounds | | NA | NA | NA | NA | NA | **3** (17) | **8** (44) | **3** (17) | **5** (28) |

1 Direction of gene expression effect in the previously performed large-scale human dietary intervention study investigating the chemopreventive effect of blueberry-apple juice ([9-11](#_ENREF_9))

2 Extracts were incubated at maximal concentration for 2 and 24 hours

3 X = no significant change in gene expression effect

T2, T6, T24, or T48: significant gene expression effect at time point 2, 6, 24 or 48 hours of exposure

Bold visualizations indicate similar direction of gene expression effect in the previously performed large-scale human dietary intervention study as compared to the gene expression effects in Caco-2 cells after pre-incubation with the extract of blueberry apple juice, the extracts of four different blueberry varieties, and/or four individual phytochemicals

4 Individual phytochemicals were incubated at 50 µM for 2, 6, 24 and 48 hours
