# Supplementary figures and images for: Improved Preventive Effects of Combined Bioactive Compounds Present in Different Blueberry Varieties as Compared to Single Phytochemicals
Source: Nutrients. 2018 Dec 29;11(1):61. doi: 10.3390/nu11010061 (PMC6356906; doi:10.3390/nu11010061)

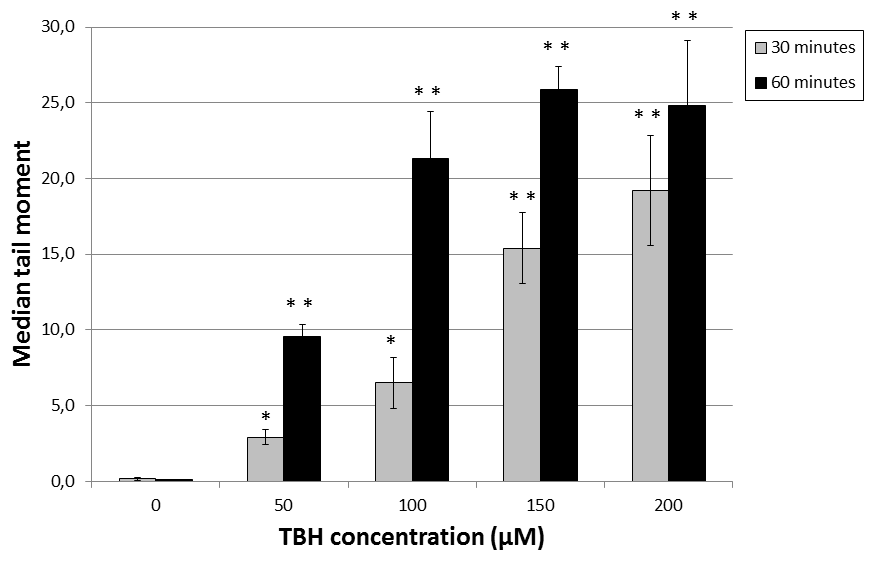

Supplement: Supplementary file 1 [file nutrients-11-00061-s001.zip › Supplementary Figure 1.tif]

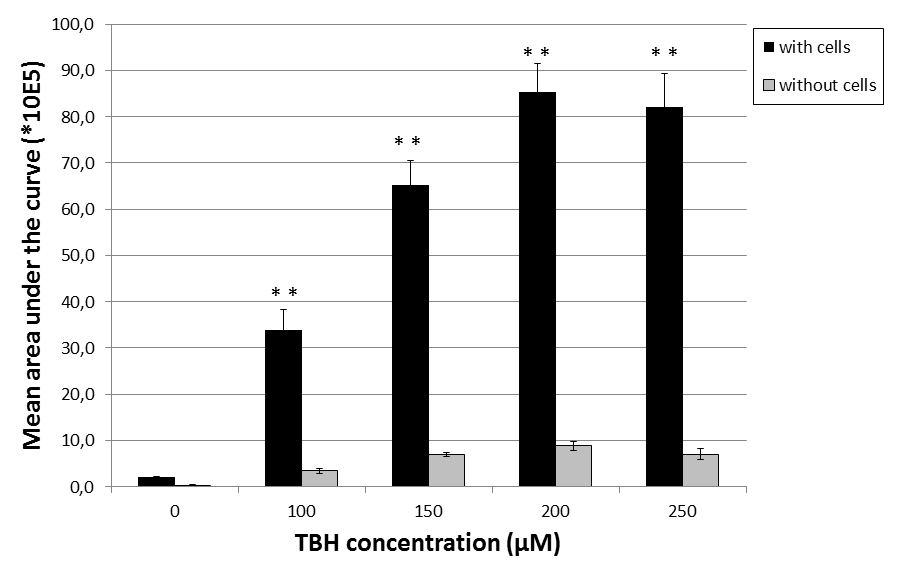

Supplement: Supplementary file 1 [file nutrients-11-00061-s001.zip › Supplementary Figure 2.tif]
